# Esophageal Cicatricial Pemphigoid as an Isolated Involvement Treated with Mycophenolate Mofetil

**DOI:** 10.1155/2015/620374

**Published:** 2015-10-18

**Authors:** Sandra Sánchez Prudencio, Daniel Domingo Senra, Daniel Martín Rodríguez, Belén Botella Mateu, Carlos Esteban Jiménez-Zarza, Felipe de la Morena López, José Jiménez Reyes, Manuel Nevado Santos, Beatriz de Cuenca Morón

**Affiliations:** ^1^Gastroenterology Department, Hospital Infanta Cristina, Avenida 9 de Junio, No. 2, 28981 Parla, Madrid, Spain; ^2^Dermatology Department, Hospital Infanta Cristina, Avenida 9 de Junio, No. 2, 28981 Parla, Madrid, Spain; ^3^Pathology Department, Hospital Infanta Cristina, Avenida 9 de Junio, No. 2, 28981 Parla, Madrid, Spain

## Abstract

Cicatricial pemphigoid (CP) is a rare blistering autoimmune disease. Esophageal involvement occurs in widespread disease and rarely appears as the only affected organ. We report a 67-year-old Caucasian female with esophageal dysphagia and weight loss. Several oral panendoscopies showed multiple exudative ulcerations with fibrin and webs in mid- and proximal esophagus and a peeling mucosa. There were no lesions in other organs. We established the diagnosis performing a direct immunofluorescence (DIF), demonstrating IgG3 and complement deposition along the basement membrane. As initial treatment the patient received prednisone 60 mg and 1 gr twice daily of mycophenolate mofetil (MMF) as a steroid-sparing agent due to its lower toxicity and its selective mechanism of action. Six months later there was a significant clinical improvement and the esophageal ulcerations had disappeared, developing cicatricial fibrous rings, although no stenosis was present. Four years later, the patient remains asymptomatic with a low maintenance dose of MMF.

## 1. Introduction

Cicatricial pemphigoid (CP), also known as mucous membrane pemphigoid, is a rare blistering autoimmune disease that primarily affects mucous membranes of the oropharynx, conjunctiva, and genitals and less frequently the skin [1, 2]. Esophageal involvement occurs in widespread disease and rarely appears as the only affected organ. In these cases the disease usually has an aggressive course, even with esophageal stenosis requiring dilations. So it is important that an aggressive treatment with corticotherapy and immunosuppressor drugs be prescribed from the beginning [3].

We report the second case of CP with only esophageal lesions and the first case treated with prednisone and mycophenolate mofetil (MMF) without disease symptoms during four years of evolution.

## 2. Case Report

**Figure 1:**
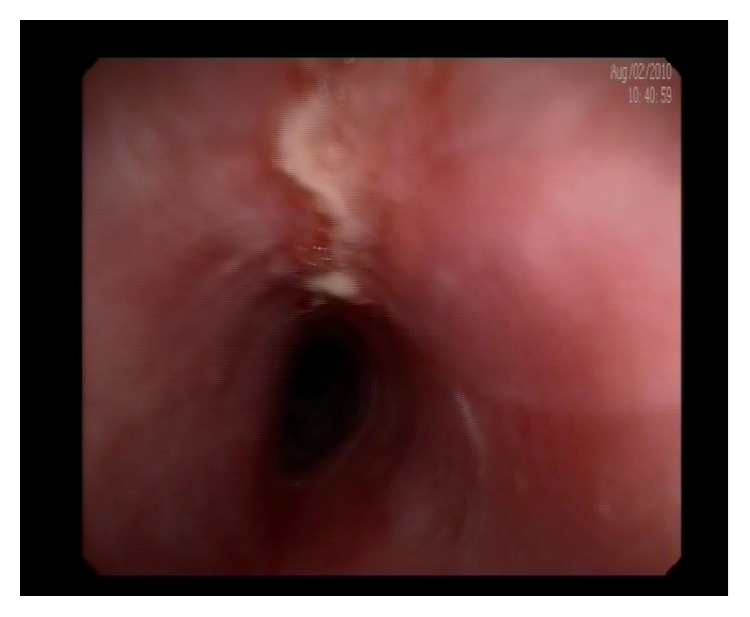
Esophageal ulcerations.

**Figure 2:**
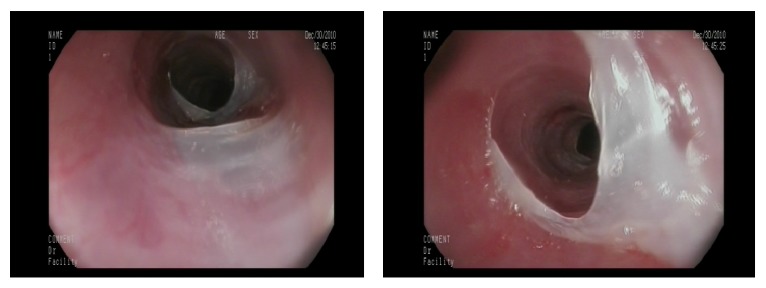
Esophageal webs in mid- and proximal esophagus.

**Figure 3:**
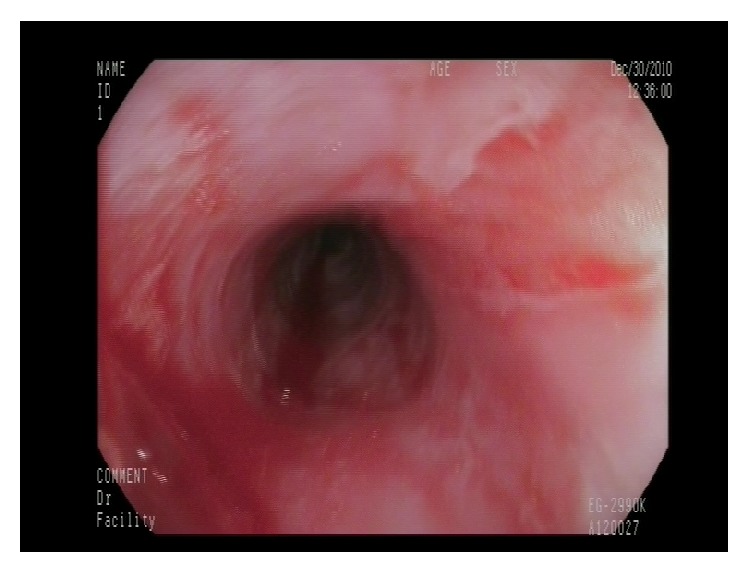
Peeling esophageal mucosa.

**Figure 4:**
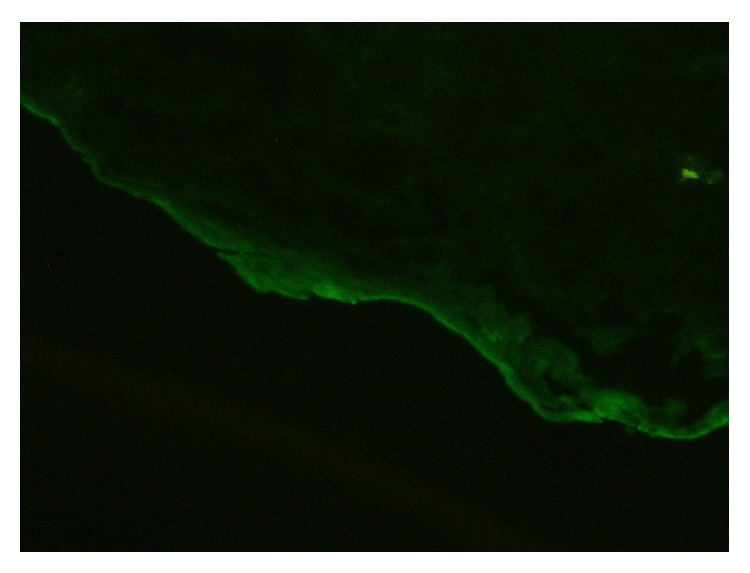
IgG deposition along the basement membrane.

**Figure 5:**
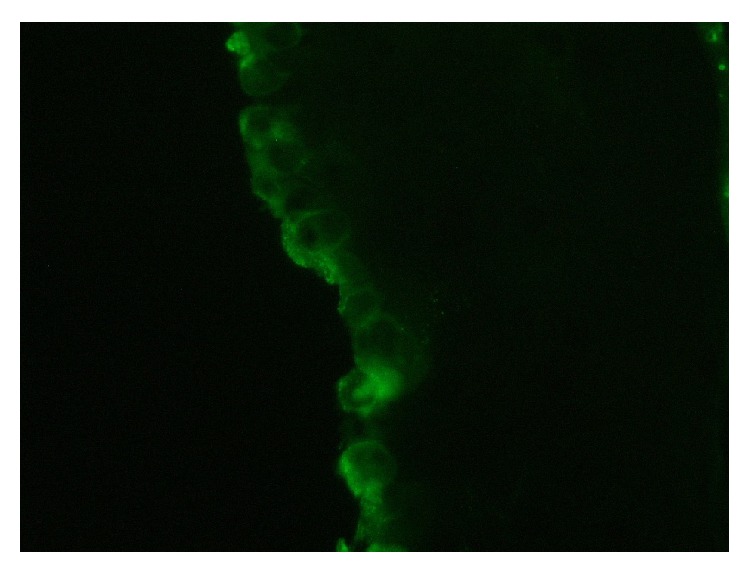
C3 deposition along the basement membrane.

**Figure 6:**
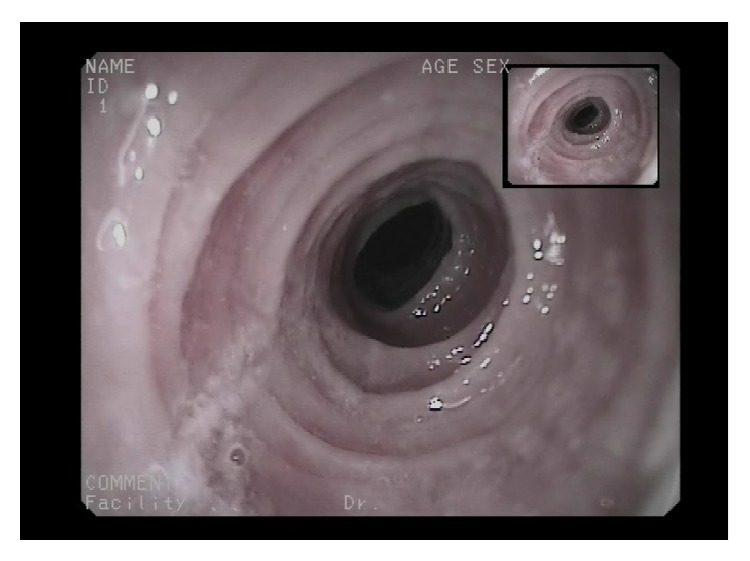
Six-month treatment later: cicatricial fibrous rings, no stenosis.

We present a 67-year-old female with high blood pressure and previous ictus with no sequelae. She was being treated with antiaggregant (acetyl salicylic acid). She complained of two-year evolution of intermittent dysphagia for solids, not associated with chest pain or significant weight loss. A high resolution esophageal manometry and 24-hour pH-metry were found to be normal. An oral panendoscopy showed multiple exudative ulcerations with fibrin (Figure 1) and webs in mid- and proximal esophagus (Figure 2) and a peeling mucosa (Figure 3). Initially we suspected pill-induced esophagitis so that the acetyl salicylic acid (ASA) intake was stopped and antiacid was prescribed, but the patient did not improve. Several oral panendoscopies (OPE) were carried out with similar findings discarding peptic and viral esophagitis, candidiasis, and even HIV. An esophagogram did not show esophageal stenosis and a thoracic-cervical CT was also normal. We performed diagnosis of exclusion with the possibility of esophageal pemphigoid cause of esophageal ulcers. Therefore we performed a biopsy with direct immunofluorescence (DIF), demonstrating IgG and C3 complement deposition along the basement membrane (Figures 4-5). There were no ocular or skin lesions, only vulvar erythema with no pemphigoid signs on the biopsy. As initial treatment the patient received prednisone at 1 mg/kg daily (60 mg) and 2 gr daily of MMF as a steroid-sparing agent. Six months later there was a significant clinical improvement: the patient had no dysphagia and had gained weight. Moreover the esophageal ulcerations had disappeared, developing cicatricial fibrous rings, although no stenosis was present (Figure 6). This allowed us to use MMF maintenance dose of 500 mg daily. The control gastroscopies showed no evidence of reactivation of the disease. Four years later the patient continues asymptomatic, without other extraesophageal manifestations.

## 3. Discussion

CP is a chronic autoimmune disease and occurs in middle-aged patients and more commonly in women, with an incidence of 1/100000 annually. It presents like subepithelial blisters basically in mucosa, which progress to scar and fibrosis. Oropharynx is the most common site, presenting as gingivitis or palate, tongue, and lip lesions. Also ocular lesions are frequent; in fact CP can be limited to the eyes, starting with an insidious form as a chronic conjunctivitis, but causing more serious illness like entropion, symblepharon, ankyloblepharon, or even blindness. Other locations are nasopharynx, larynx, esophagus, and genitals [1, 4]. Skin lesions are infrequent and they are usually mild. Esophageal involvement is rare, with an incidence of 3%, and normally appears in disseminated disease [5–8]. Interestingly, this may be present up to 10 years after the initial onset of the disease [9, 10]. In the first stage we can observe webs and ulcerations and in the last phase fibrotic rings that inevitably develop into stenosis. PC diagnosis requires a high index of suspicion, mainly if the oesophagus is the only affected organ [10]. In this case we needed several attempts until biopsy perilesional ulcer from esophagus was obtained for DIF. This is the most sensitive and specific test to CP diagnosis. In the vast majority of patients (80–100%) it displays a linear, continuous band along the basement membrane zone of IgG (predominantly IgG4), C3, and occasionally IgA. DIF using salt split improves its sensibility. The indirect immunofluorescence (IIF) shows circulating anti-basement membrane zone IgG antibodies (or IgA) at low titles [1, 11]. Some cases require other techniques for diagnosis such as the immunochemical test or immunoelectron microscopy. Histologic examinations show subepithelial blisters with an underlying superficial inflammatory infiltrate containing lymphocytes, histiocytes, and eosinophils, but these findings are nonspecific [12]. Esophageal PC management depends on clinical presentation and the severity of the symptoms. Oral systemic corticoids are the most commonly used in advanced illnesses, specifically prednisone 1 mg/kg, with good short term [5] results. However, systematic glucocorticoids are often associated with immunosuppressors like azathioprine or cyclophosphamide as steroid-sparing agents [7, 13, 14]. But these treatments can cause significant side effects such as marrow aplasia, infections, neoplasia, or hemorrhagic cystitis. MMF is an immunosuppressive drug that selectively inhibits T and B lymphocytes blocking the de novo purine synthesis. Doses of 2–2,5 gr/d represent a promising therapy due to their low toxicity and their selective mechanism of action. In fact, there are some reported clinical cases treated with MMF successfully [15]. Esophageal PC usually cause esophageal stenosis that needs repeated dilations [3, 4, 7, 16]. In most cases this technique achieves good results but it entails a high risk of perforation [3]. All patients require long term monitoring because PC is a chronic and recurrent disease. Our patient was initially treated with 60 mg/d of prednisone with descendent dosage and MMF 2 g/d (1 g/12 hours). Two months later we practiced an OPE, demonstrating improvement of the lesions and six months later there was complete recovery of ulcerations and she was asymptomatic for dysphagia and without any bullous lesions in other organs. After clinical and endoscopic improvement we maintained dose of 500 mg MMF daily indefinitely to prevent recurrence of disease and complications like esophageal strictures and to avoid possible PC involvement of other organs. There is only one reported case in the literature of isolated esophageal involvement [13], treated with cyclophosphamide instead of MMF with marked initial symptomatic response, but with short term follow-up. We decided not to use cyclophosphamide due to its potential adverse effects. MMF was administered based on the good response observed in several published clinical cases that primarily treated pemphigoid with ocular involvement with MMF. All of them reported good response and minimal adverse effects [17–19].

Esophageal PC is a rare condition and its natural history and prognosis are not completely known [1].

This is the second case with only esophageal affectation after four years of follow-up and the first treated with MMF with good evolution.

We recommend to make DIF of perilesional mucosa in patients with multiple esophageal erosions without clear etiology.
